# Bilateral Continuous Thoracic Paravertebral Block for the Pain Management of Multiple Rib Fractures With Flail Chest: A Case Report

**DOI:** 10.7759/cureus.75406

**Published:** 2024-12-09

**Authors:** Shota Tanimoto, Tomoharu Shakuo, Takuya Dosei, Atsunori Sakamoto, Kenji Shida

**Affiliations:** 1 Anesthesiology, Showa University Northern Yokohama Hospital, Yokohama, JPN

**Keywords:** anesthesia, catheterization, flail chest, multiple rib fractures, paravertebral block

## Abstract

Flail chest is a life-threatening condition characterized by multiple rib fractures that result in a partially free rib cage. Thoracic paravertebral block (TPVB) allows visualization of the needle tip under ultrasound guidance and can be safely performed, unlike epidural anesthesia where the needle tip cannot be visualized. Here, we describe a case of flail chest in whom TPVB was used, as it provides the same level of analgesia as epidural anesthesia and has a perfect analgesic effect. A 58-year-old man with multiple rib fractures and a flail chest underwent emergency sternal fixation under general anesthesia with postoperative bilateral TPVB and catheter placement. The left side was difficult to puncture and thus required puncture twice. After TPVB, the patient was returned to the intensive care unit under intubation. On postoperative day 2, the catheter on the left side leaked a large amount of fluid and was removed. The patient was extubated on postoperative day 3. The right catheter was removed on postoperative day 5. The patient was discharged at 14 days postoperatively without complications. The patient reported no significant postoperative pain. TPVB may be a useful option with analgesic effects and reduced circulatory depression, particularly if epidural anesthesia cannot be performed with a flail chest.

## Introduction

Flail chest is a life-threatening condition characterized by multiple rib fractures that result in a partially free rib cage [1]. Traumatic rib fractures inhibit physiological thoracic motion, cause severe pain, and may result in atelectasis and pneumonia. Rib fracture treatment primarily aims to prevent pneumonia by reducing pain and improving the respiratory status [2]. Surgical rib fusion is an effective treatment for multiple rib fractures and flail chests [1]. Previously, conservative treatment was the mainstream approach; however, prolonged artificial ventilation is problematic because of the high incidence of pneumonia, tracheotomy, and pressure trauma, which significantly reduce the patient's quality of life [3]. Ultrasound-guided thoracic paravertebral block (TPVB) addresses the spinal and sympathetic nerves and can be safely performed under general anesthesia.

We report a case of traumatic rib fracture wherein epidural anesthesia was difficult to administer preoperatively in an emergency surgery. The patient had severe pain due to the fractures and had unstable oxygenation. Thus, administering epidural anesthesia was deemed difficult preoperatively. After general anesthesia, the patient was in an unstable hemodynamic state, receiving continuous intravenous phenylephrine and circulatory agonists to maintain hemodynamics. Circulatory stabilization was thus prioritized over nerve block. Therefore, we decided to manage the pain with bilateral continuous TPVB postoperatively.

## Case presentation

**Figure 1:**
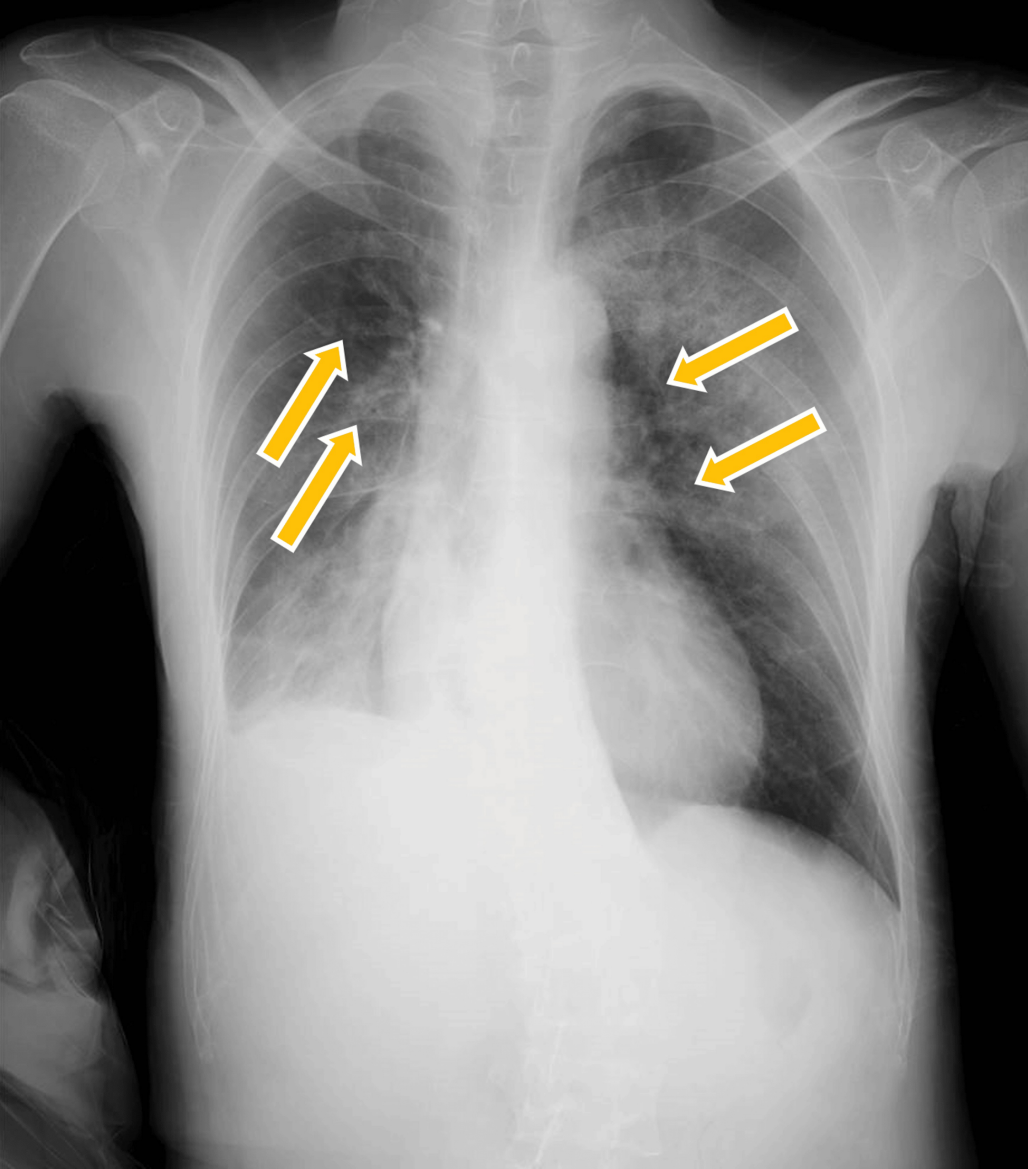
Preoperative chest radiograph. The orange arrows indicate multiple fractures of the ribs

**Figure 2:**
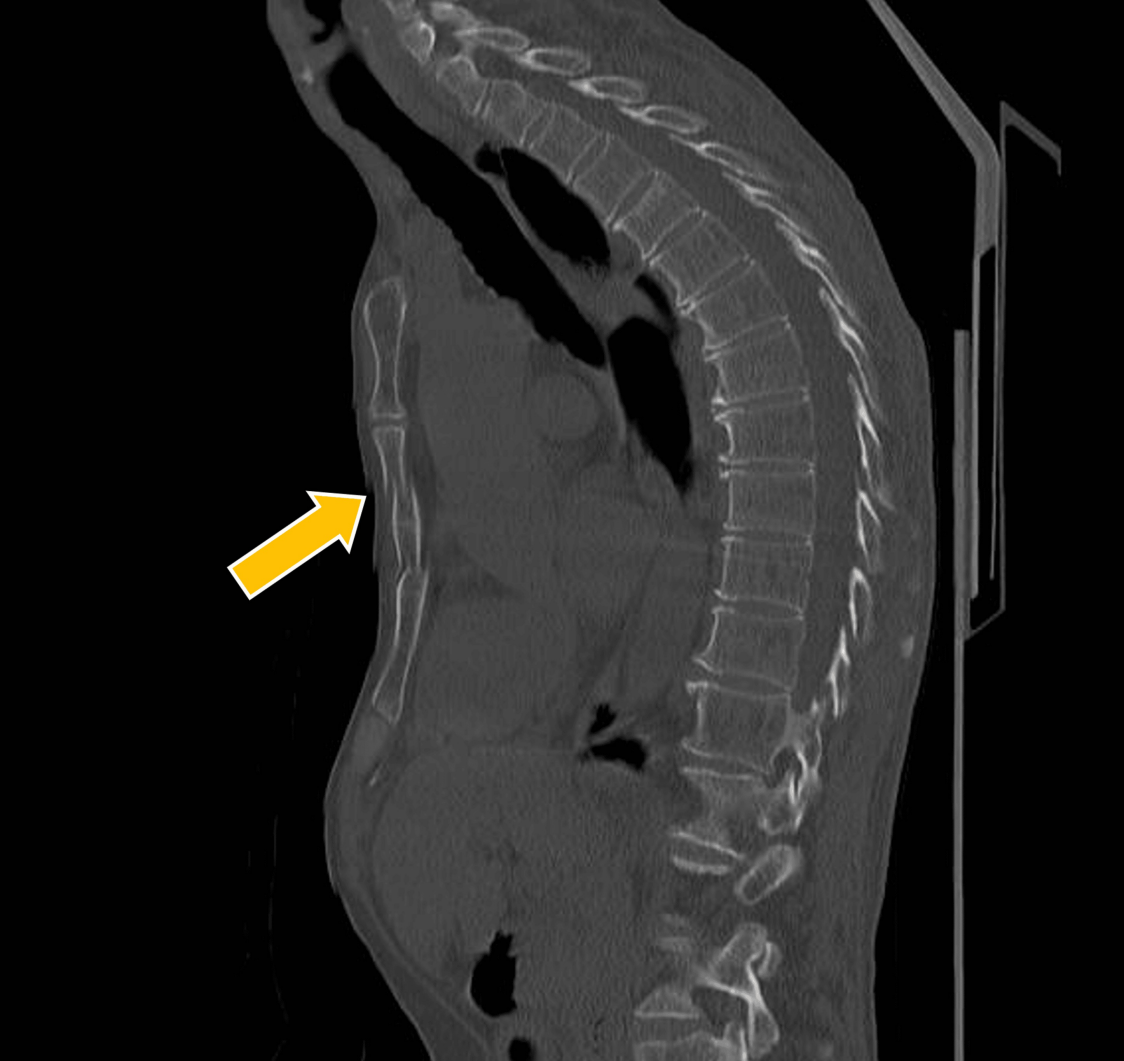
Preoperative chest computed tomography. The orange arrow indicates fracture of the sternum

**Figure 3:**
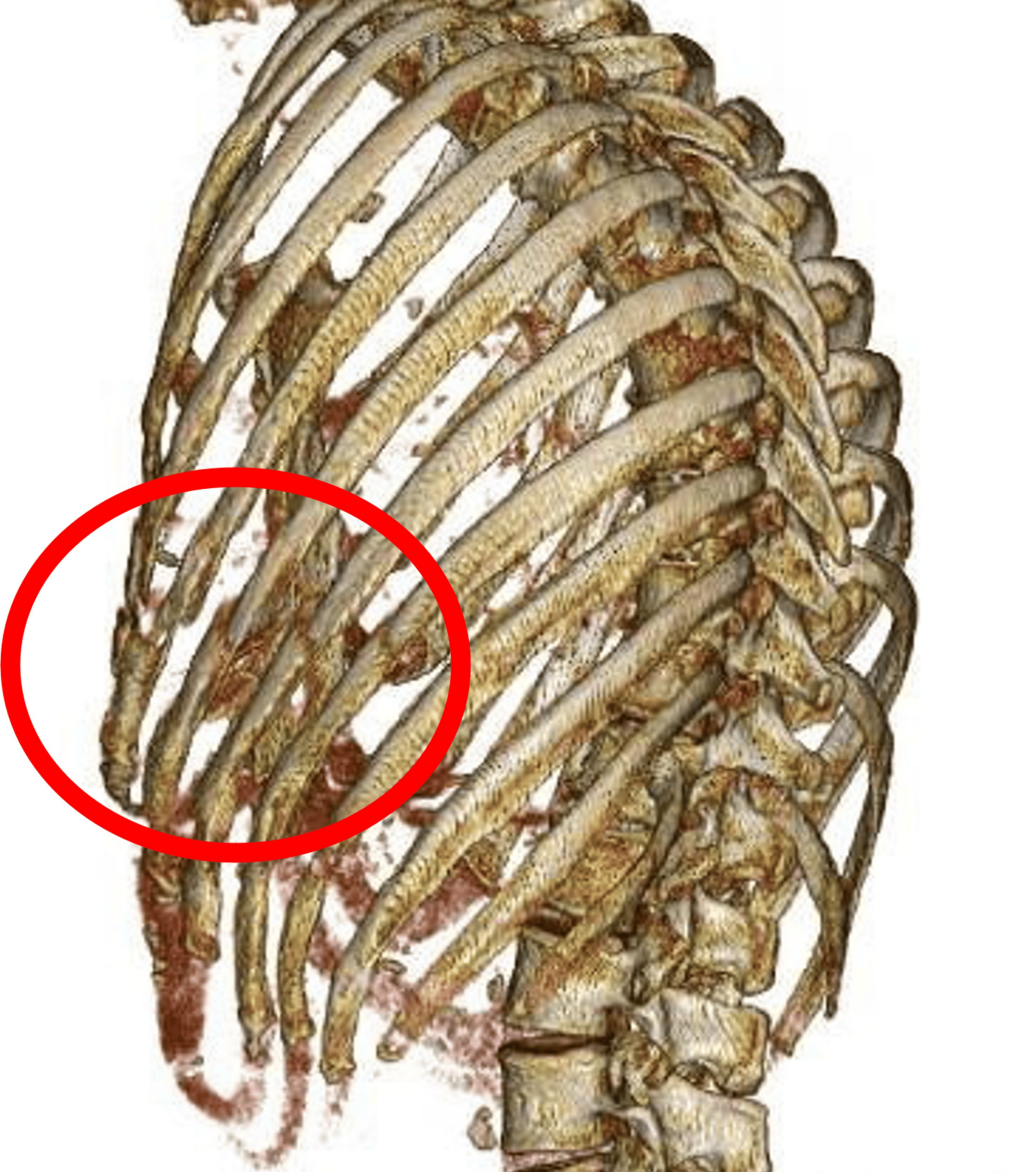
Preoperative three-dimensional chest computed tomography. The red circle indicates multiple rib fractures on the left at the level of the fifth to eighth thoracic vertebrae

**Figure 4:**
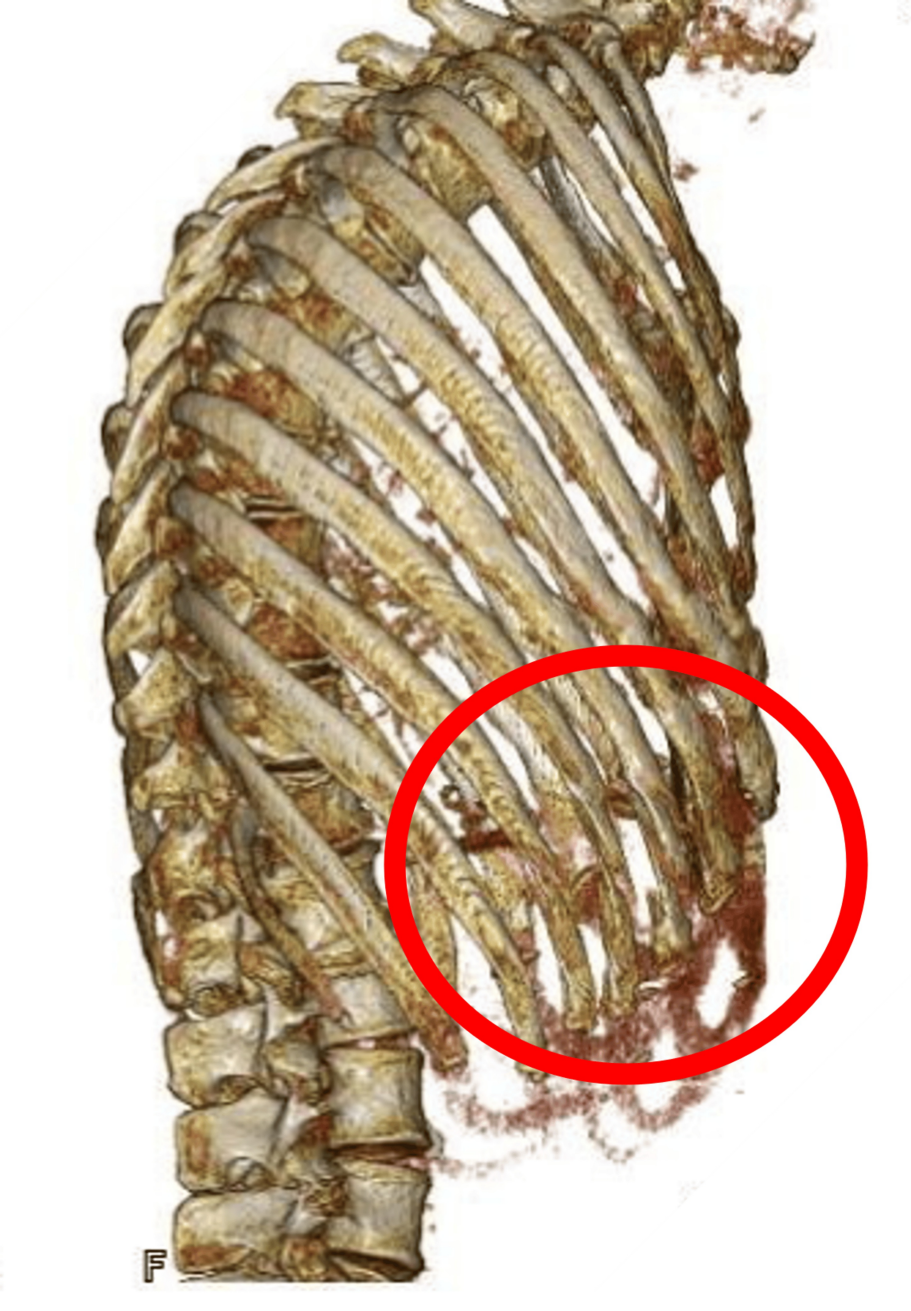
Preoperative three-dimensional chest computed tomography. The red circle indicates multiple rib fractures on the right at the level of the seventh to ninth thoracic vertebrae

A 58-year-old man (height 168 cm, weight 45 kg) stumbled at home and hit his rib cage hard on a chair. He had a history of funnel chest, varicose veins in both lower extremities, and childhood asthma, and was not taking any medication. Imaging revealed multiple fractures of the sternum and ribs (Figures 1, 2), with multiple rib fractures on the left at the level of the fifth to eighth thoracic vertebrae and on the right at the level of the seventh to ninth thoracic vertebrae (Figures 3, 4). The patient was diagnosed with multiple rib fractures and flail chest.

The patient’s vital signs on admission were as follows: blood pressure of 187/114 mmHg, heart rate of 79 beats/minute, respiratory rate of 30 breaths/minute, and arterial blood oxygen saturation of 94% (oxygen mask with a reservoir of 15 L/min). His oxygen requirement gradually increased, prompting a decision to perform emergency sternal fixation. Physical examination revealed that the thorax was depressed on inspiration and distended on expiration, indicating flail chest.

General anesthesia with peripheral nerve blockade was administered. Anesthesia was rapidly induced with propofol, remifentanil, and rocuronium. Maintenance was achieved using air, oxygen, and desflurane. The patient had severe pain due to the fractures and demonstrated unstable oxygenation. Thus, administering epidural anesthesia was deemed difficult preoperatively. Consequently, we decided to manage the pain with bilateral continuous TPVB postoperatively.

**Figure 5:**
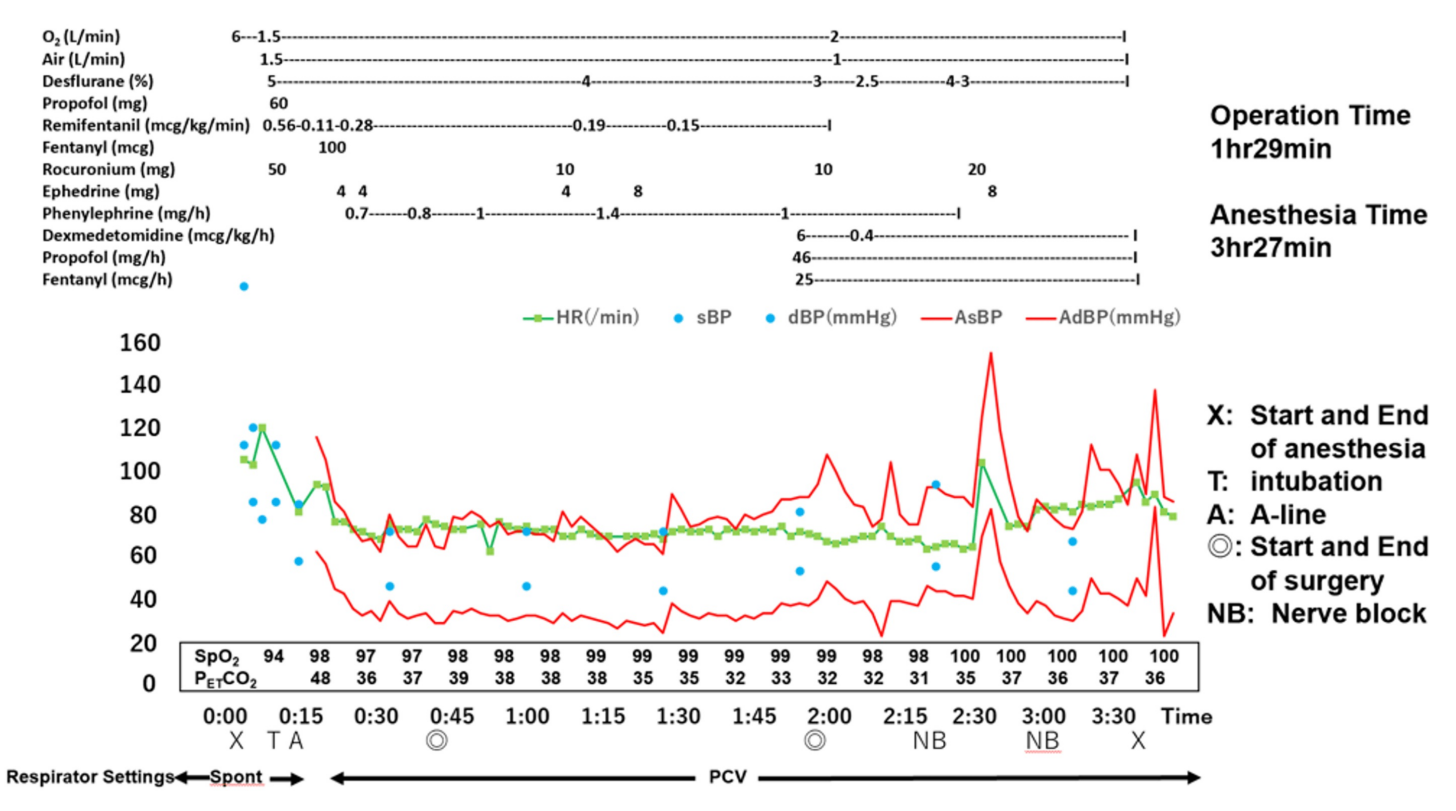
Anesthetic record

**Figure 6:**
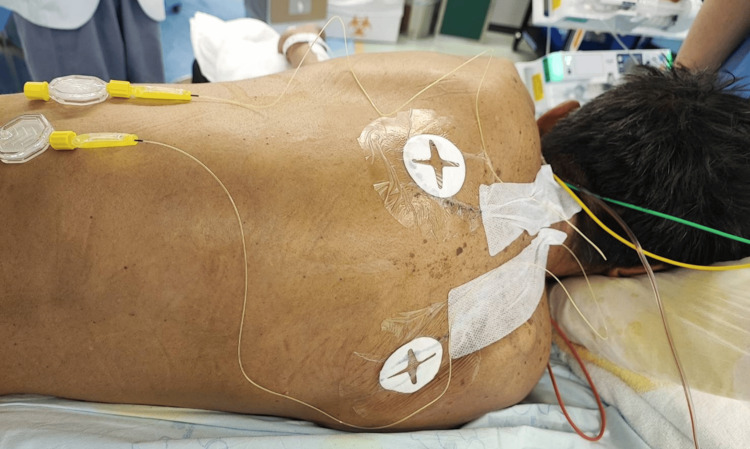
Posterior view of a patient in the lateral decubitus position Implanted bilateral Th4/5 Perifix® ONE catheters were used. Also, 2-Ethyl cyanoacrylate (Aron Alpha®) film dressing was used for fixation.

After oral intubation and positive-pressure ventilation, his arterial blood oxygen saturation improved to approximately 97%. The ventilator was set to a controlled pressure, the fraction of inhaled oxygen was maintained at 0.5, and surgery was completed without any complications (Figure 5). At the time of wound closure, 1000 mg of acetaminophen and 50 mg of flurbiprofen was injected intravenously. Postoperatively, TPVB was performed at the bilateral fourth to fifth thoracic levels under ultrasonographic guidance (SonoSite SII™, FUJIFILM, Tokyo, Japan). After injecting 25 mL of 0.25% levobupivacaine into the paravertebral space on each side, a catheter for continuous peripheral nerve block (Perifix® ONE Catheter, B BRAUN, Melsungen, Germany) was fixed through a needle at a 5-cm insertion distance on both sides and 7 cm subcutaneously. For fixation, 2-ethyl cyanoacrylate (Aron Alpha®, West Jefferson, OH, USA) and film dressing were used (Figure 6). After catheter placement, 0.125% levobupivacaine 6 mL/h was continuously administered bilaterally. Continuous nerve block was provided in a COOPDECH Balloonjector™ (Daiken Medical Instruments Co., Ltd., Osaka, Japan) with a total volume of 300 mL (150 mL each of 0.25% levobupivacaine and saline solution). The left side was difficult to puncture and thus required puncturing twice.

**Figure 7:**
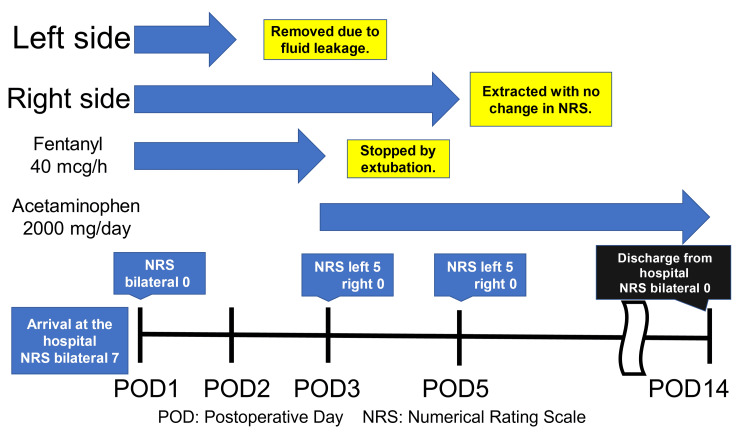
Postoperative record.

After TPVB, the intubated patient returned to the intensive care unit (ICU), with propofol (1 mg/kg/h), dexmedetomidine (0.48 μg/kg/h), and fentanyl (0.9 μg/kg/h) administered continuously. The wound numerical rating scale (NRS) was 0/10 bilaterally. On postoperative day 2, the left catheter leaked a large amount of fluid and was removed. The patient was extubated on postoperative day 3. The NRS was 0 at rest and 5 during body movement on the left side and 0 at rest and 0 during body movement on the right side. Continuous intravenous fentanyl was discontinued following extubation, and regular oral acetaminophen (2000 mg/day) was started. The right catheter was removed on postoperative day 5, and the patient was discharged on postoperative day 14 (Figure 7).

## Discussion

This report described a case of flail chest for which TPVB was used, as it provides the same level of analgesia as epidural anesthesia, blocking pain completely.

Multiple rib fractures are the most common blunt trauma, accounting for 43% cases [4], resulting in mortality rates of up to 51% [2]. TPVB has been found to be as effective as epidural anesthesia for postoperative analgesia for cases with multiple rib fractures, in whom adequate chest analgesia is required, to promote coughing and deep breathing to reduce respiratory complications [5]. Regarding anesthesia management, lung recruitment techniques should also be performed as appropriate to avoid postoperative atelectasis.

Analgesia by opioid management has historically been the first choice for multiple rib fractures. Although opioids alone provide adequate analgesia, they can cause complications, such as sedation, respiratory depression, cough suppression, and delirium [6]. Early administration of nonsteroidal anti-inflammatory drugs (NSAIDs) reportedly decreases opioid requirement, pneumonia incidence, length of hospital stay, length of ICU stay, and number of ventilator days [7]. Combined use of acetaminophen and NSAIDs may reduce opioid use, decrease the risk of complications, and increase analgesic effects [6].

Recently, a combination of intercostal nerve block, epidural nerve block, TPVB, intrapleural local narcotic infusion, intravenous patient-controlled analgesia, and oral opioids has been reported as a method of analgesia after chest surgery [8]. However, intercostal nerve blocks have a localized analgesic effect and must be injected above and below the affected ribs to achieve adequate analgesic coverage. Multiple punctures are required, increasing the risk of vascular puncture and pneumothorax [6]. Epidural analgesia may also cause complications such as hypotension [9]. Coagulation disorders are often associated with severe trauma, and in unstable pelvic fractures and spinal trauma, the position of the patient may be restricted, often making the procedure contraindicated or impossible [10]. TPVB targets the paravertebral space, a wedge-shaped cavity formed by the anterior surface of the anterolateral pleura, posterior surface of the superior transverse costovertebral ligament, medial surface of the vertebral body, and intervertebral foramen [11]. This space contains branching spinal nerves, sympathetic nerve fibers, and intercostal vessels embedded in fatty tissue and is usually continuous at the level of the thoracic spine [11]. When a local anesthetic is administered in this space, the drug diffuses superiorly and inferiorly along the vertebral bodies and blocks the spinal and sympathetic nerves. Compared with epidural anesthesia, TPVB has reduced effects on cardiac rhythm and fewer complications [9]. Previous reports have concluded that its analgesic effect is equivalent to that of epidural anesthesia [12] and that continuous TPVB reduces respiratory complications and mortality [13]. In our case, continuous TPVB provided adequate analgesia without complications.

In thoracic surgery, the following relatively new nerve blocks are considered for postoperative analgesia: TPVB, erector spinae plane block (ESPB), serratus anterior plane block (SAPB), intercostal nerve block (ICNB), and intertransverse process block (ITPB). A previous report [14] described using ESPB for multiple rib fractures, which provided analgesia equivalent to that of thoracic epidural anesthesia and may also better stabilize circulatory dynamics than epidural anesthesia. However, in a report examining postoperative morphine consumption in thoracoscopic surgery, the consumption decreased in the following order: TPVB > ESPB > ICNB > SAPB [15]. In breast cancer analgesia, ESPB is reportedly less effective than TPVB for postoperative analgesia due to the higher postoperative morphine consumption [16]. Despite its greater technical difficulty, TPVB is expected to provide better postoperative analgesia (Table 1).

**Table 1:** Comparison of nerve blocks. +Low. ++Moderate. +++High. ESPB, erector spinae plane block; FRC, functional residual capacity; TEA, thoracic epidural analgesia; TPVB, thoracic-paravertebral block

	TPVB	TEA	ESPB
Ease	Difficult	Moderate	Easy
Accuracy	++	+++	+
Risk	++	++	+
Benefit	Better pulmonary function and fewer pulmonary complications than TEA [9].	Excellent pain relief. Improvement in respiratory parameters including FRC and lung compliance [6].	Hemodynamic stability. Risk of hemorrhage or hematoma is very low [14].
Complications	Same as epidural analgesia but less frequent than with TEA [9].	Hypotension, urinary retention, nausea, and vomiting [9].	Unable to predict how far local anesthetics will spread [14].

ITPB is another block that has received attention for thoracic analgesia. Administering local anesthetic at the back of the superior transverse thoracic ligament (retro-SCTL) blocks the spinal and sympathetic nerves. The retro-SCTL is widely connected to the paravertebral space, intervertebral foramen, and erector spinae, and partially to the intervertebral foramen, which is directly connected to the paravertebral space via the medial and lateral slits of the superior transverse costal ligament. Retro-SCTL and the adjacent paravertebral space are connected by loose connective tissue, termed the costotransverse space, and adipose tissue, which act as conduits [17,18]. Therefore, the ITPB is expected to have similar analgesic effects as the TPVB, as the drug administered in the retro-SCTL reaches the paravertebral space by passing through the medial and lateral slits of the superior transverse costovertebral ligament and the costotransverse space. Moreover, use of ITPB during breast-conserving surgery reduced the amount of postoperative opioid use [19]. Thus, ITPB may also be useful for chest analgesia, including for multiple rib fractures.

## Conclusions

Pain management in cases with multiple rib fractures and flail chest may benefit from bilateral continuous TPVB when epidural anesthesia is difficult to perform, as in our case. TPVB may provide similar analgesic effects as epidural anesthesia, but with less circulatory depression and provides excellent analgesia.
